# Enhanced Data Mining and Visualization of Sensory-Graph-Modeled Datasets through Summarization

**DOI:** 10.3390/s24144554

**Published:** 2024-07-14

**Authors:** Syed Jalaluddin Hashmi, Bayan Alabdullah, Naif Al Mudawi, Asaad Algarni, Ahmad Jalal, Hui Liu

**Affiliations:** 1 School of Computing, National University of Computer and Emerging Science, Islamabad 44000, Pakistan; i212237@nu.edu.pk; 2 Department of Information Systems, College of Computer and Information Sciences, Princess Nourah bint Abdulrahman University, P.O. Box 84428, Riyadh 11671, Saudi Arabia; bialabdullah@pnu.edu.sa; 3 Department of Computer Science, College of Computer Science and Information System, Najran University, Najran 55461, Saudi Arabia; naalmudawi@nu.edu.sa; 4 Department of Computer Sciences, Faculty of Computing and Information Technology, Northern Border University, Rafha 91911, Saudi Arabia; asaad.algarni@nbu.edu.sa; 5 Faculty of Computing and AI, Air University, E-9, Islamabad 44000, Pakistan; 6 Cognitive Systems Lab, University of Bremen, 28359 Bremen, Germany

**Keywords:** sensors datasets, Bio–Mouse–Gene, data visualization, big data, data mining, graph summarization, weighted LSH, correction sets

## Abstract

The acquisition, processing, mining, and visualization of sensory data for knowledge discovery and decision support has recently been a popular area of research and exploration. Its usefulness is paramount because of its relationship to the continuous involvement in the improvement of healthcare and other related disciplines. As a result of this, a huge amount of data have been collected and analyzed. These data are made available for the research community in various shapes and formats; their representation and study in the form of graphs or networks is also an area of research which many scholars are focused on. However, the large size of such graph datasets poses challenges in data mining and visualization. For example, knowledge discovery from the Bio–Mouse–Gene dataset, which has over 43 thousand nodes and 14.5 million edges, is a non-trivial job. In this regard, summarizing the large graphs provided is a useful alternative. Graph summarization aims to provide the efficient analysis of such complex and large-sized data; hence, it is a beneficial approach. During summarization, all the nodes that have similar structural properties are merged together. In doing so, traditional methods often overlook the importance of personalizing the summary, which would be helpful in highlighting certain targeted nodes. Personalized or context-specific scenarios require a more tailored approach for accurately capturing distinct patterns and trends. Hence, the concept of personalized graph summarization aims to acquire a concise depiction of the graph, emphasizing connections that are closer in proximity to a specific set of given target nodes. In this paper, we present a faster algorithm for the personalized graph summarization (PGS) problem, named IPGS; this has been designed to facilitate enhanced and effective data mining and visualization of datasets from various domains, including biosensors. Our objective is to obtain a similar compression ratio as the one provided by the state-of-the-art PGS algorithm, but in a faster manner. To achieve this, we improve the execution time of the current state-of-the-art approach by using weighted, locality-sensitive hashing, through experiments on eight large publicly available datasets. The experiments demonstrate the effectiveness and scalability of IPGS while providing a similar compression ratio to the state-of-the-art approach. In this way, our research contributes to the study and analysis of sensory datasets through the perspective of graph summarization. We have also presented a detailed study on the Bio–Mouse–Gene dataset, which was conducted to investigate the effectiveness of graph summarization in the domain of biosensors.

## 1. Introduction

A graph, consisting of vertices and edges, depicts the insights of biological networks [1]; this can be useful in various applications, such as in the online health community (for summarizing data on certain diseases, like diabetes [2]), in hyperlink networks [3], in social networks [4], in cooperation networks [5], in citation networks [6], in road networks [7], in shared purchasing networks [8], in producing dependency graphs for biomedical relation extraction [9], and in the internet of medical things [10] among others. Growth in the usage of these aspects has led to an increased research interest in the underlying network science and its analytics. The usage of graphs and other relevant datasets are also of great interest to researchers in the fields of graph mining [11,12], neural networks [13,14,15,16,17], graph neural networks [18,19,20,21,22], deep learning [23,24,25,26,27], data mining [28,29], and machine learning [30,31]. Researchers around the globe are using these datasets to gain insights into complex systems, allowing them to make informed decisions [32]. Biosensing is a state-of-the-art area of research that involves studies on the text mining of health documents [33], chemical sensing [34], medical imaging [35,36], brain age prediction [23], food safety [37], and biosensors [38], among others. Exploring these domains by modeling their datasets as graphs is also a valuable research avenue [23,39,40,41,42]. A graph or set of graphs can be used to represent some of the aforementioned aspects; these comprise large numbers of nodes and edges, and they continuously expand at a remarkable pace. These real-world graphs are too large to fit in the main storage of software; yet, answering complex queries on them in real time requires them to be readily available [43]. Therefore, it is vital to represent them in a concise manner which is efficient and scalable [44,45,46].

**Figure 1:**
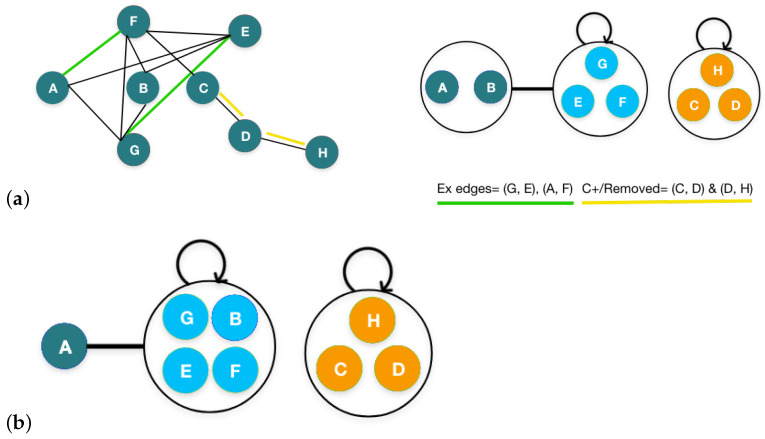
Illustration of grouping-based and personalization graph summarization. (**a**) Correction-set-based summarization. (**b**) Personalization graph summarization.

Compressing graphs into a compact form is useful when working with them in various scenarios such as storing, processing, querying, and visualizing. In this situation, a potential solution is graph summarization, which generates a more compact representation, called a summary graph, of the input graph. A summary graph reduces the footprint of the original graph and facilitates efficient query answering and insightful data visualization. There have been a number of research studies for graph summarization [47]. The group-based approach is one of the most popular methods for graph summarization [45,48]. This group-based approach takes a simple, undirected graph as input and produces a summary graph and a correction set. The objective of summarization in this line of work is to minimize the size of the summary graph and correction sets while preserving all information from the original graph. The summary contains the member nodes of the input graph, which are merged into super nodes based on certain criteria, and correction sets are used to reconstruct the original graph. SWeG is a correction-set-based graph summarization algorithm that has been presented recently [44]. It is fast, yields high compression, and can run in a distributed setting as well. It adds a dividing step that splits nodes into smaller groups before merging them, making the algorithm more efficient and parallelizable, as shown in Figure 1a. It introduces an approximation metric for identifying nodes to merge. Overall, SWeG aims to improve the performance and accuracy of the graph summarization process. However, there are performance bottlenecks in the merging and encoding algorithms that affect their efficiency for larger graphs. The merging phase is an issue because some groups can be extremely large, leading to longer running times. Additionally, the approximation method used for selecting similar nodes for grouping can result in lower compression rates. Some of the inefficiencies of SWEG [44] are improved by a state-of-the-art algorithm named LDME (Locality-sensitive hashing Divide Merge and Encode) [45]. It proposes weighted locality-sensitive hashing, can handle large datasets on a single machine, and can balance compression and running time. It achieves a significant speedup with similar or better compression than SWeG and up to two orders of magnitude of acceleration, but there are reductions in compression.

An important point to note for the above-mentioned approaches is that they are general graph summarization methods, i.e., they perform the merging of the nodes without focusing on the importance of certain highlighted nodes in the network [5,46]. For instance, certain member nodes of a graph have distinct levels of engagement or inclination towards the particular elements or features of a graph. Consequently, the significance of designing visual representations that are customized to meet the needs of the intended viewers and successfully communicate the intended message is underscored. Consider the following scenarios: social media users are more interested in the connections of their close acquaintances rather than those of strangers; travelers prioritize the roads in their vicinity rather than those further away; researchers are more interested in the papers related to their field than those in other fields. This highlights the importance of tailoring network visualizations to suit the specific interests and needs of a given target audience. In this regard, personalized graph summarization (PGS) is an effective approach [43] that takes individual preferences into account during summarization. In PGS, given a large graph and a target node, for personalized summary graph generation, the objective is to obtain a summary that merges the rest of the nodes while considering the existence of target nodes. This algorithm ensures that the resulting summary accurately reflects the preferences and requirements of the target node, as shown in Figure 1b. However, one drawback of PGS is that it is not an efficient algorithm when applied on large-sized graph datasets. We can be motivated by the fact that the graph data from the aforementioned domains contain useful relationship patterns; however, they are huge in size, so it is not easy to mine for information in order to discover knowledge and ensure its visualization. This issue is non-trivial and of high value when graph data represent interactions among living beings, like the data from the fields of healthcare, disease research, and bioengineering, among others.

In this regard, we present a new algorithm that produces a summary graph having similar compression ratio to that of state-of-the-art algorithm (PGS [43]), but it is more efficient when it is applied to large graph datasets. In this way, we aim to provide faster data mining and analysis techniques for experts in this field to support them in studying the problems at hand from a different perspective. We name our algorithm IPGS and propose the concept of weighted locality-sensitive hashing (LSH) for the personalized summarization of an input graph. The proposal of weighted LSH enhances the efficiency of the algorithm, particularly for handling high-dimensional data, where LSH has proven to be more effective. Our algorithm can handle large-sized graph datasets effectively on a single machine. Finally, the proposed approach is lossless, since we maintain a list of correction sets which are also computed in an optimized manner. We present the effectiveness analysis and performance comparisons of our approach on eight real-world datasets—including the Bio–Mouse–Gene dataset which has 43.1 K nodes and 14.5 M edges—and derived better results for execution time in comparison with the PGS [43]. We also perform experiments for the evaluation of the compression that can be achieved in comparison with the current state-of-the-art algorithm for non-personalized graph summarization, LDME [45]; this is highly scalable but was found to be less effective in terms of providing less compression. To make our research widely usable, we release the implementation of our proposed approach, IPGS, along with the implementation of PGS [43] and LDME [45] on https://github.com/jalal-gilgiti/IPGS (accessed on 9 July 2024). We summarize the contributions of our paper below to clearly demonstrate our work.

- Given the large sizes of graph datasets, we have proposed an efficient algorithm named IPGS for graph summarization. The algorithm models locality-sensitive hashing to locate similar nodes for compression. The proposed algorithm produces a similar compression ratio as that of a state-of-the-art algorithm but is less time-consuming.
- The proposed algorithm provides a lossless summary graph through the concept of a correction set. This is beneficial since we can always reconstruct the original one or use the correction set for querying the result with 100% accuracy.
- We performed detailed experimental evaluation on eight real-world and publicly available datasets and provided insightful results by comparing with two state-of-the-art approaches. We also present a detailed study on the Bio–Mouse–Gene dataset to demonstrate the usefulness of our approach and the concept of graph summarization in the domain of biosensors.

## 2. Literature Review

In this section, we review the existing studies addressing the different topics of biosensors and graph summarization.

### 2.1. Review of Knowledge Discovery Techniques in Biosensors and Multidisciplinary Domains

In this section, we review various studies in the disciplines of biosensors, bioengineering, and other relevant fields; this is because data mining and knowledge discovery in the fields of health informatics, biosensors, and cross-domain research is presently one of the most active areas of research. In particular, researchers from numerous areas of computer science and artificial intelligence have investigated these areas from the perspective of their own expertise. In this regard, we witness researchers in data mining [49,50,51], machine learning [52,53], pattern mining [54], data compression [55,56], decision support [57,58,59,60], and visualization [61,62,63] producing insightful knowledge and actionable information.

The contribution of decision-support systems to the field of bioengineering [57,59,60] is of massive value. These systems serve as a backbone of a one-window platform for various types of data storage, information retrieval, knowledge discovery, and inference, prediction, and analytic purposes. In this regard, the authors of [60] presented a feature selection-based prediction model for dental care. They used an ensemble of decision trees as the core machine learning model for the task and obtained significantly higher classification performances. Similarly, the authors of [59] present a decision-support system for glaucoma treatment. The dataset included details of demographics, a history of systemic conditions, medication history, ophthalmic measurements, 24-2 VF results, and thickness measurements from OCT imaging, involving around 900 patients. They applied several machine learning algorithms to the data obtained from independent and geographically separated populations and obtained very promising results. On a similar note, the authors of [57] developed a decision-support system to facilitate the prediction of COVID-19 diagnosis; this used the clinical, demographic, and blood marker data of a given patient. They collected the dataset from a hospital in India and applied machine learning and deep learning algorithms for classification purposes. One of the notable contributions of their work is their focus on explainable AI; this means that the end users of the system are able to understand the type of results they obtain and understand why they should believe the results. Estimation of crop yield [64], prediction of sick leave [65], image compression [66], and rice plant disease classification [67] are further examples of the versatile applications of machine leaning techniques.

Graph neural networks (GNNs) comprise another useful approach in advanced machine learning. Researchers have utilized them for various useful applications, like urban region planning [18], summarizing vast amount of text data [19], point-of-interest recommendation [20], human activities recognition [21], and music recommendation [22]. In [18], the authors made use of random forests with CNNs for the purpose of urban planning by modeling the data in the form of a graph. The authors in [19] used GNN for text summarization. Using the concept of graphs, they are able to model the relationship between words, and accurately extract feature information and eliminate redundant information as well. Similarly, the authors in [20] used the same technique for another interesting aspect of finding suitable points of interest for people; this can help in providing appropriate customer matches for merchants. Similarly, music recommendation [22] and humans action recognition in healthcare [21] are wonderful areas of research focus.

Studying the behavior of large-scale biological networks for pattern discovery is of key importance [54], where the authors introduce a innovative model for the degree of distribution of nodes in the network. Normally, the degree of distribution of numerous real-world networks/graphs exhibit power-law degree distribution. In this regard, the contribution of this research is enormous: they provide a versatile distribution model to provide new insights. The authors of [37] review various research studies on the topic of food safety in the context of biosensing. They studied various analytes, like glucose, gluten, gliadin, atrazine, domoic acid, arsenic, and various others, in their research to address the control of food quality and safety; the aforementioned types of data are studied in detail. Analyzing them by modeling them as ontologies is also worthwhile. An ontology—for instance, for genes data [39]—represents a comprehensive view of the underlying data whose inference provides useful insights. Similarly, using wearable sensors in the research and development of biosensing information systems is of value [38]. This system provides a multidimensional view of the collected data for the betterment of healthcare.

Considering the aforementioned brief review of the various studies from the biosensors and bioengineering domain, we find that a number of researchers have explored machine learning and AI techniques for problem solving purposes in remote sensing [38,68,69,70,71,72] and image processing [73,74,75,76,77]—among other versatile areas [78,79,80,81,82] and multidisciplinary fields [83,84,85,86,87]—and have contributed significantly.

### 2.2. Review of Research on Graph Summarization

Graph summarization is a widely explored research domain encompassing diverse methodologies for effectively summarizing graph datasets [47,88,89,90,91,92]. The purpose of all of these studies is to reduce the size of the input graph so that it can be effectively and efficiently mined in the pursuit of knowledge discovery and visualization. To perform summarization, the existing methodologies include both group-based and non-group-based approaches. Among them, the group-based approach is particularly prominent. This can be further classified into cohesive correction-set-based and non-correction-set-based approaches. Notably, correction-set-based approaches have garnered greater attention, owing to their remarkable compression and summarization outcomes. Consequently, we have chosen to employ a correction-set-based approach as the foundation for our study.

The correction set approach has led to the development of various algorithms for graph summarization, including VOG [93], Mosso [94,95] DGPS, SSumM [32], SWEG [44], PGS [43], SAGS [48], and LDME [45], among others. These algorithms use different methodologies in various domains to summarize graphs. VOG [93] is a lossless graph summarization technique that determines whether large graphs consist of various sub-graphs, such as cliques, stars, and chains. Each sub-graph type contains distinct information and has a significant impact on the entire graph. It is crucial to understand the information contained within sub-graphs and measure them based on their importance for decision making. The above-mentioned study solved the following vital question: how can we measure the significance of sub-graphs within large graphs? SSumM is a similar lossless summarization algorithm that produces a sparse summary graph [32]; it uses the minimum description length (MDL) principle—as does [96], which is a pioneering work in this field. SSumM identifies important structures within large graphs and develops efficient methods for their summarization and visualization. The authors of [94] present lossless incremental summarization to preserve the information of the dynamic changes that have been made to the graph, such as the addition or deletion of edges. SAGS [48] is a similar correction-set-based approach to the summarization of large graphs. It models LSH [97] to locate sets of similar nodes for compression. The non-mergeable nodes in a given iteration in a located set are pruned out based on their dissimilarity from the rest of the nodes. The algorithm proposed in [95] also makes use of the degree of the nodes during the summarization process. It aims to preserve the degree of each node in the summarized graph for better graph processing, storage, and analytics.

SWEG [44] is a useful correction-set-based algorithm for graph summarization that consists of three steps: merging nodes to super nodes, encoding edges to super edges, and dropping edges for compact graph representation. It provides better compression than previous algorithms and improves the existing frameworks by adding a dividing step before merging the nodes; this divides them into disjoint groups for parallel processing. Additionally, it introduced the approximation metric to achieve the best match for merging. However, the SWEG algorithm’s performance is impacted by certain steps. For instance, the merging algorithm has a quadratic running time due to the identification of disjoint groups, which can affect its speed. The authors of [98] leverage the MDL principle to provide intuitive, coarse-level summaries of input graphs while effectively managing the errors. Additionally, there have been efforts to refine existing techniques to enhance their performance, particularly concerning densification procedures [45]. Improvements in densification aim to address the issues that are related to randomness and accuracy, particularly in sparse datasets, which are common on the web. Through theoretical analysis and experimental evaluations, these enhancements demonstrate their superiority over previous schemes, particularly for very sparse datasets.

On the other hand, it may be be noted that the aforementioned correction-set-based approaches primarily perform non-personalized graph summarization. With the escalating size of data, people are presently displaying greater interest in extracting relevant information from big data. Taking user preferences into account, researchers have developed personalized graph summarization algorithms which aim to achieve the summarization of a large graph from the point of view of the input/target nodes [5,43,46]. VEGAS [5] stands out as one of the pioneering algorithms for personalized graph summarization; it is specifically designed for citation networks. It is important to highlight that this algorithm solely focuses on citation networks. Another state-of-the-art algorithm in this domain is personalized graph summarization (PGS) [43], which employs greedy search techniques. Finally, the algorithm in [46] also proposes an efficient, weighted LSH-based algorithm for personalized graph summarization; thus, it is unlike VEGAS [5], which is quite effective but is very slow when applied to large-sized graphs.

## 3. Problem Statement

In this age of advanced technology, large-sized datasets from various disciplines—like data for brain signals, medical topics, vital signs, medical text, biomedical signals, sensors, and social networks—are available for research and innovation purposes. In this context, our goal in this research is to efficiently generate a summary of a large-sized input graphs, so that meaningful analysis can be performed in-memory and more effectively. Formally, we take a dataset modeled in the form of a graph, *G*, having vertices, *V*, representing entities from a corresponding domain, and edges, *E*, showing interactions among the entities. Taking this, we aim to develop a scalable algorithm to summarize *G* into a compact representation of a summary graph, $G^{/}$, where those vertices that have similar properties can be merged into super nodes, $V^{/}$, and their corresponding edges can be merged as super edges, $E^{/}$. In particular, we want to have an efficient algorithm where the $G^{/}$ is obtained from the point of view of the user-provided target nodes (s), in order to obtain a personalized summary graph. Our $G^{/}$ is lossless since we maintain a correction set, C+, C−, where C+ is a list of edges which are removed while merging certain vertices and C− is a list showing the edges that are added during aggregation. In this way, we aim to generate a compact-sized $G^{/}$, with minimized $V^{/}$+ $E^{/}$ + C+ + C−, based on the MDL principle [96].

In this regard, PGS [43] provides an algorithm for personalized graph summarization which provides a highly compressed summary; however, it is not scalable when it is applied to large graph datasets. Our aim in this research is to improve its execution time by providing a similar compression ratio. By incorporating this improvement, our new algorithm, IPGS, is highly scalable, and it provides an accurate and comprehensive summary; thus, it meets the diverse needs and requirements of users.

## 4. The Proposed Algorithm, IPGS

In this section, we present our proposed approach in detail. Our approach is a lossless summarization due to the concept of correction set attached to the summary graph; so, we first present the steps of how to perform the merging of the nodes of a *G* while maintaining a list of corrections. We then explain the inside details of IPGS, followed by the formal algorithm for the summary generation.

### 4.1. Correction-Set-Based Approach for Grouping-Oriented Summarization

**Figure 2:**
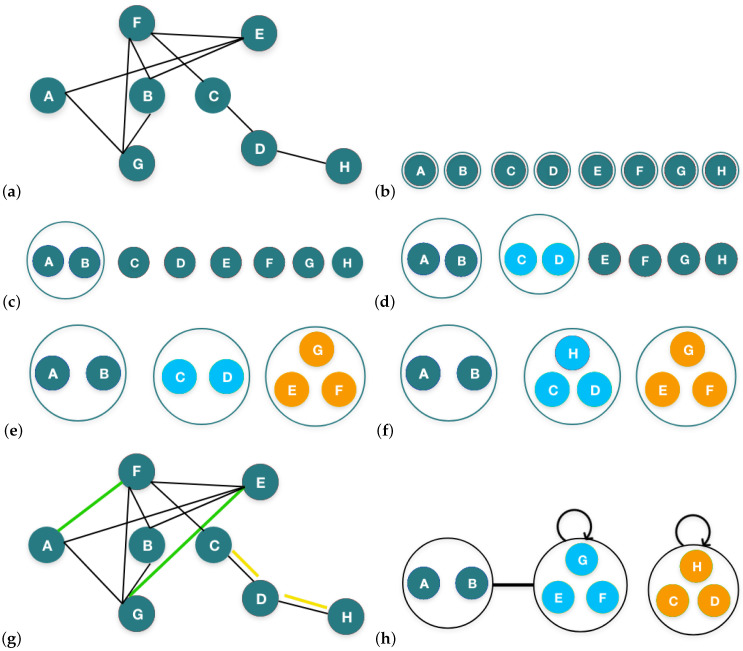
Illustration of correction-set-based merging: (**a**) input graph *G*; (**b**) initialization phase. (**c**) merging iteration 1; (**d**) merging iteration 2; (**e**) merging iteration 3; (**f**) merging iteration 4; (**g**) representation of C+ (yellow) and C- (green) edges; (**h**) final summary graph $G^{/}$.

We take an undirected graph *G* as input, having vertices *V* and edges *E*, as shown in Figure 2a. In this illustration, the algorithm iterates four times. As a first step, each individual node is called a super node. The algorithm then updates every super node by merging the nodes in each iteration based on the maximum saving produced by the merger. The merging process reduces the sum of the super edges *P* along with positive C+ and negative C− edge corrections, denoted as $\left(E^{/}\right)+\left(C+\right)+\left(C-\right)$. The formula to calculate the savings obtained from merging two nodes, 1 and 2, i.e., A and B in Figure 2a, is shown in Equation (1).

(1) $$\mathrm{Saving}=1-\frac{\mathrm{Cos}t(1\cup 2)}{\mathrm{Cos}t(1,S)+\mathrm{Cos}t(2,S)}$$

where Cost(1, S) and Cost(2, S) are the contributions of nodes 1 and 2 in $\left(E^{/}\right)+\left(C+\right)+\left(C-\right)$. In this way, the merging is repeated for a certain number of iterations by randomly selecting a super node then finding a node to be merged with it; this provides the highest savings using Equation (1). This process is repeated once all of the super nodes are merged.

The original edges *E* from *G* are then encoded into the super edges and the correction sets. During the encoding of the edges, we encounter two different sets of edges, i.e., the original edges between the super nodes and the total number of possible edges between them. The number of the original edges is represented by EAB and the possible edges are represented by FAB. To perform edge encoding for a pair of super nodes, A and B, if the number of the original edges is less than or equal to half of the possible edges between them, then one does not encode the edge and instead adds the original edge to C+. On the other hand, if the number of original edges is greater than half of the possible edges between them, then one should encode the super edge and add the extraneous edges to C−. Additionally, the two super nodes, A and B, are further merged as a new super node.

### 4.2. Weighted LSH for IPGS

The previous section explains the various steps involved in the merging of nodes in producing a general-purpose summary. One of the bottlenecks is a result of the issue of how we can efficiently identify the nodes providing the highest savings in each iteration, while preserving the personalization aspect during the summarization process. To solve these problems, we modelled LSH to ensure that similar nodes are grouped together. LSH speeds up the node identification process by approximating the Jaccard similarity among the nodes and hashes the similar nodes in groups. LSH groups similar nodes by employing a hash function or a set of hash functions on the neighborhood structure of each node. This hashing process pushes similar nodes to the same buckets.

**Figure 3:**
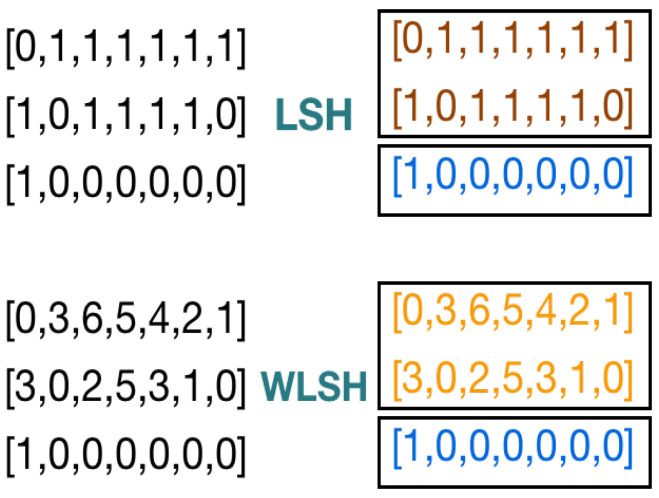
LSH and WLSH applied on node A, B, and G of input graph *G*.

We now illustrate the concept of weighted Jaccard similarity, denoted as $J_{w}\left(A,B\right)$, on to the neighborhoods of two nodes *A* and *B*. Their neighbors are represented as vectors of equal length and comprise integer weights, as demonstrated in Figure 3. The definition of this weighted Jaccard similarity is as follows in Equation (2):

(2) $$J_{w}\left(A,B\right)=\frac{\sum_{v}\min\left(A_{v},B_{v}\right)}{\sum_{v}\max\left(A_{v},B_{v}\right)}$$

**Figure 4:**
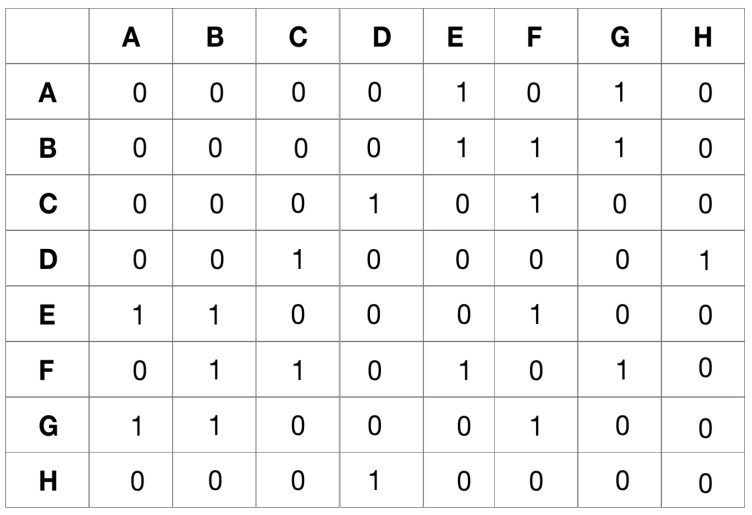
Adjacency matrix of input graph in Figure 2.

**Figure 5:**
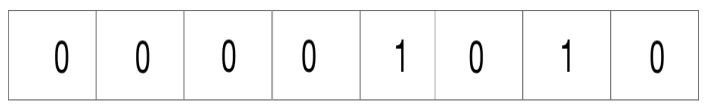
Applying DOPH on Node A.

**Figure 6:**
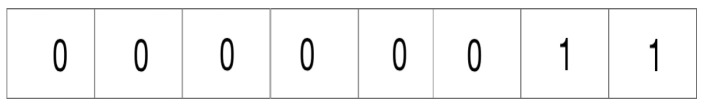
Permute the vector using random permutation-based hash function *H*.

**Figure 7:**
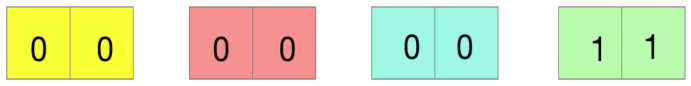
Divide the permuted vector into *K* equal bins; here, *K* is signature length chose by user and is assumed as 4.

**Figure 8:**
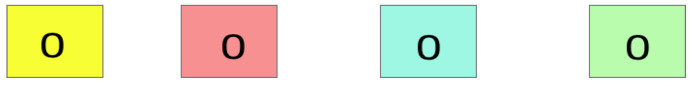
If bi has a non-zero entry, set Hbi as index of the first non-zero entry; otherwise, let bi be the first bin on the left or right with a non-zero entry; set Hbi to Hbj.

**Figure 9:**
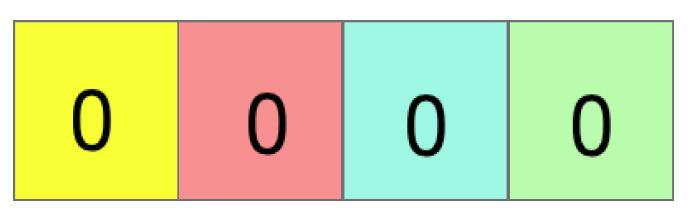
Hash signature for node A.

To model LSH for weighted Jaccard similarity, we use the concept of densified one permutation hashing (DOPH) [99]. To generate a hash signature for a node in DOPH, we start with a binarized vector, denoted as *I*. We then shuffle the elements of vector *I* using a chosen hash function to create a permuted vector. The next step is to determine the desired length for the hash signature and divide the permuted vector *I* into equal bins based on this length. From each bin, the first non-zero value is selected. In a case where there are no non-zero values in a bin, consider a value from either the left or right neighboring bins. Finally, the resulting hash signature is returned. We illustrate this process in Figure 4, Figure 5, Figure 6, Figure 7, Figure 8 and Figure 9.

To utilize the concept of personalization, we aim to preserve the influential impact and flow patterns within the summarized graph, ensuring that the essential dynamics of aggregation with respect to target node *T* is retained. By employing this approach, the goal is to retain and accurately represent the influential flow patterns within the summarized graph. This ensures that the resulting summary effectively captures and preserves the influential dynamics of the original data. For illustration purposes, let us consider the weighted variant of our input sample graph to generate a summarization comprising *k* clusters and *l* flows, while maximizing an objective function that incorporates cumulative flow rates, as show in Equation (3).

(3) $$\max\left(\sum_{s=1}^{l}r\left(\xi_{s}\right)\right)$$

**Figure 10:**
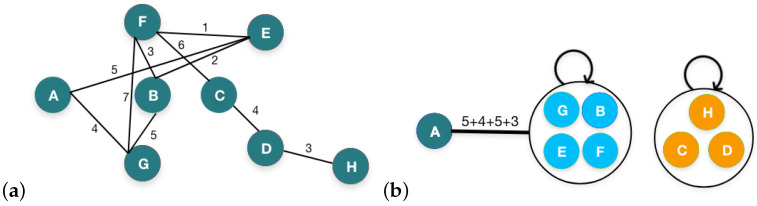
Illustration of IPGS based on flow rate maximization: (**a**) input-weighted graph; (**b**) output summary graph maintaining influence of node A.

In this example, we have nodes with weights assigned to their edges. To perform personalized graph summarization based on flow rate maximization, we take the weighted edges into account, while identifying the influential flow patterns within the graph. We consider both the strength of the connections and the flow of influence or information between nodes. In the given weighted graph in Figure 10, let us assess the flow rates considering node A as the target node. The various flow rates are as follows: 5 from A to E, 4 from A to G, 9 from A to B through G, and 12 from A to F through G and B. These flow rates indicate the influence of node A on the flow of information or interactions within the graph. Based on the flow rates and connections, we can summarize the graph to represent the essential pathways of influence from node A. The summarized graph includes node A, along with the merged nodes that receive the highest flow rates directly from node A and through other nodes as the immediate neighbors.

In the summarized graph in Figure 10, we capture the primary most influential pathways from node A to nodes E, G, and also B and F, with moderate flow. This representation focuses on preserving the most significant connections and flow rates originating from node A while providing an overview of the impact of node A on the graph. It is important to note that the summarized graph for the impact of node A may not include all nodes and edges from the original graph. It specifically highlights the influential connections originating from node A, emphasizing the flow patterns and connections that contribute most significantly to the impact of node A on the overall graph dynamics.

### 4.3. Formal Algorithms of the Proposed IPGS

**Figure 11:**
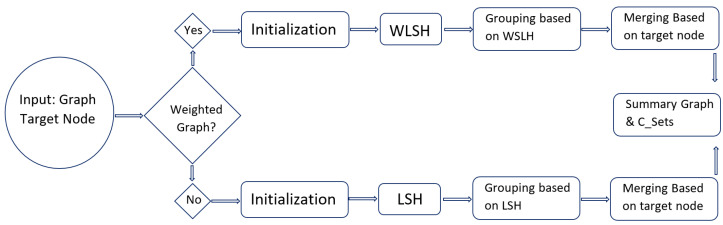
Flow diagram for proposed algorithms.

Our algorithm takes as input a graph along with a target node or set of target nodes, and the counter for the maximum number of iterations to perform. The goal is to generate a summary graph that preserves the influence or impact of the target node(s), while summarizing the original graph. The summary graph consists of super nodes $V^{/}$ and super edges $E^{/}$. The algorithm begins by initializing each node in the input graph as a super node. Then, in each iteration, LSH signatures are generated for each super node to group them into candidate groups based on the similarity of their signatures. Within each group, merges are performed to combine nodes; edges are encoded as super edges. Additionally, personalized error or correction sets are calculated to assess the impact of the summarization on the target nodes. After iterating through the specified number of times, the algorithm returns the summary graph and the correction sets to represent the summary error or any corrections made during the summarization process. Overall, this algorithm aims to effectively summarize the input graph while preserving the influence or impact of the target node(s) by employing LSH signatures, merging operations, and personalized error calculations. The flow rate maximization step is integrated within the main summarization loop. It calculates flow rates, identifies influential flow patterns, and updates the summary graph based on the identified patterns. We present both of the variants of the proposed approach, i.e., the pseudocode for the summarization with and without a focus on the preservation of the personalization aspects in Algorithms 1 and 2, respectively. We also present overall architecture of our working of the algorithms in flow diagram in Figure 11.
**Algorithm 1:** IPGS without considering the personalization aspect.
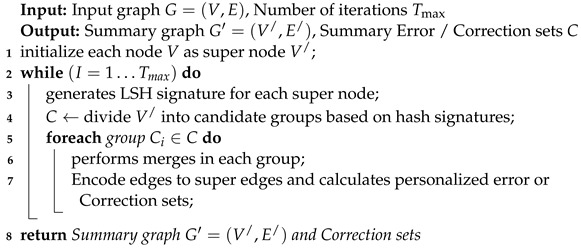


**Algorithm 2:** IPGS while considering the personalization aspect.

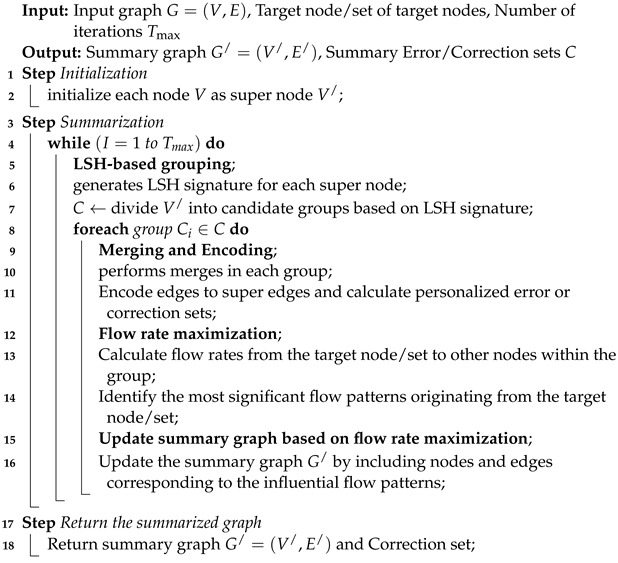



## 5. Experimental Evaluation

In this section, we present an experimental evaluation of our proposed algorithm, IPGS. We implemented the algorithms in Java language and the experiments were performed on a PC with 16 GB RAM, 250 GB SSD, and a 2.20 GHz processor. The experiments were performed to compare the execution time and compression ratio of the algorithms. We also present a detailed visualization for the Bio–Mouse–Gene dataset as a case study.

### 5.1. Data Availability

The experiments are performed on eight publicly available datasets, as listed below:

- Bio–Mouse–Gene. Nodes—43.1 K; edges—14.5 https://networkrepository.com/bio-mouse-gene.php (accessed on 9 July 2024).
- Cnr2000. Nodes—325,557; edges—5,565,380 https://networkrepository.com/cnr-2000.php (accessed on 9 July 2024).
- LastFM-Asia. Nodes—7624; edges—27,806: https://snap.stanford.edu/data/feather-lastfm-social.html (accessed on 9 July 2024).
- Caida. Nodes—26,475; edges—53,381: https://snap.stanford.edu/data/as-Caida.html (accessed on 9 July 2024).
- DBLP. Nodes—317,080; edges 1,049,866: https://snap.stanford.edu/data/com-DBLP.html (accessed on 9 July 2024).
- Skitter. Nodes—1,694,616; edges—11,094,209: https://snap.stanford.edu/data/as-Skitter.html (accessed on 9 July 2024).
- Amazon. Nodes—403,394; edges—103,310,688: https://snap.stanford.edu/data/amazon0601.html (accessed on 9 July 2024).
- Citation-Patent. Nodes—4 M; edges—17 M: https://snap.stanford.edu/data/cit-Patents.html (accessed on 9 July 2024).

### 5.2. Exploring Mouse Gene Dataset through Visualization

**Figure 12:**
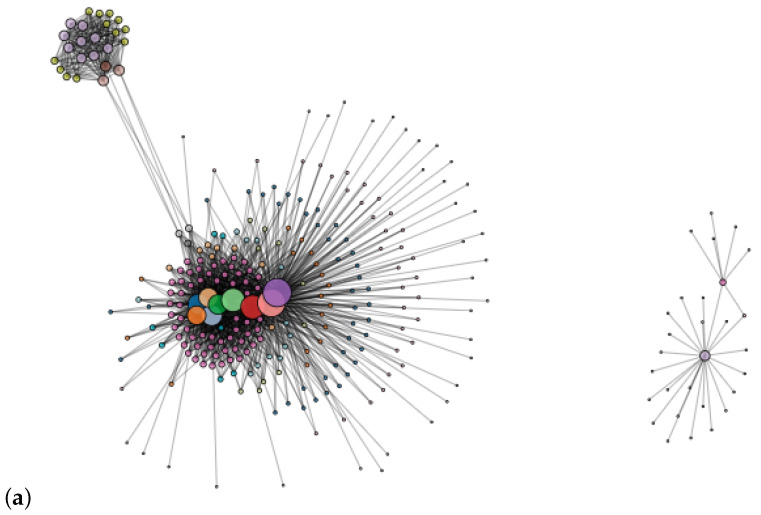

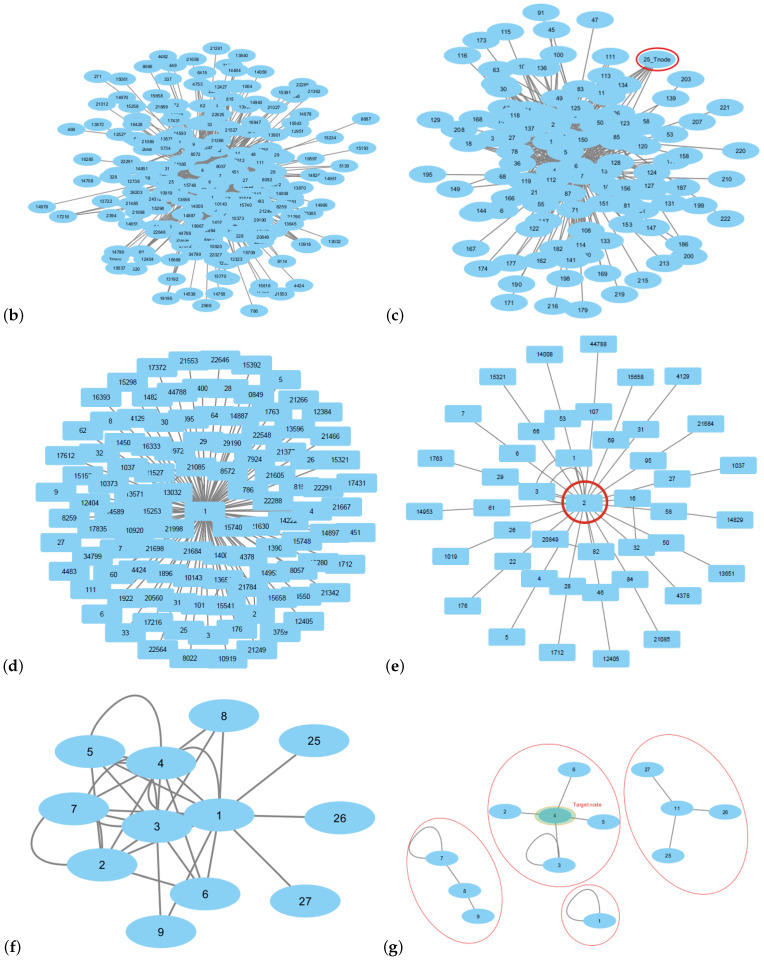
Visualization of original and summary graphs of Bio–Mouse–Gene dataset. The results are provided by varying the size of the input graph for detailed analytics and visualization. (**a**) Original Bio–Mouse–Gene dataset. (**b**) Chunk of the original dataset, having 223 nodes and 997 edges. (**c**) Summary graph, with 119 super nodes and 439 super edges, from the chunk of the dataset shown in Subfigure (**b**). (**d**) Bio–Mouse–Gene dataset with 108 nodes. (**e**) Summary graph of the graph with 108 nodes in Subfigure (**d**) for target node 2. (**f**) Smaller chunk from the original graph, with 12 nodes and 29 edges. (**g**) Summary graph, with 4 super nodes and 12 super edges, of the chunk of the dataset that is shown in Subfigure (**d**).

In this section, we show the effectiveness of our proposed algorithm, IPGS, for visualization of summarized graphs. We demonstrate this through the visualization of the Bio–Mouse–Gene dataset [40,41]. This dataset is very large in size, having 43.1 K nodes and 14.5 M edges; hence, a visualization of this entire input graph is highly cluttered, as can be witnessed in Figure 12a. We have taken this visualization from the main source of the dataset, i.e., https://networkrepository.com/bio-mouse-gene.php (accessed on 9 July 2024), for demonstration purposes. This graph is too large in size, so we have taken a chunk of it, comprising 223 nodes and 997 edges, as shown in Figure 12b; then, we generated its summary graph in Figure 12c for a target node. The target node is highlighted by a red circle. In this kind of visualization, we can inspect the target node’s impact on and relationship to its neighborhood, and to the rest of the summary graph—the tightly bonded sets of nodes that are merged with each other for a given target node. The summary graphs in the aforementioned figures are still dense and show visual clutter, so we took a smaller chunk of the dataset and visualized it in Figure 12d. This smaller chunk had 108 nodes with 110 edges; its summary graph is shown in Figure 12e. We took an even smaller chunk, comprising 12 nodes and 29 edges—shown in Figure 12f—to demonstrate a summary graph of a dataset of this smaller size. Figure 12g shows how the target node is connected to the others in each visualization.

We are using the Bio–Mouse–Gene dataset, which indicates which genes are connected to which, and how they are related to research and the study of diseases of humans. So, using this target node, depicting certain types of genes, the connected super nodes in the summary graph provide very useful insights. This knowledge is of particular interest for the exploration of new types of group-based interactions for the discovery of certain human diseases. We understand that a reader of our research cannot perform interactive analytics of the summary graphs presented in these figures because of the static nature of the images. However, by using the code and implementation shared by us, readers can run the algorithm to generate the summary graphs themselves, using any standard and latest version of graph visualization softwares, like Gephi [63] or Cytoscape [1] for an interactive analysis.

### 5.3. Comparison of Execution Time

We have performed all the evaluations on a single-threaded machine; this demonstrates the fact that the existing approaches—as well as the proposed approach—do not require much memory. We do not compare LDME for the execution time aspect because it has significantly faster performance than our proposed IPGS algorithm and the state-of-the-art PGS algorithm. Moreover, LDME is used for general-purpose grouping-based summarization applications; in contrast, PGS and IPGS can be used to perform personalized graph summarization. This is one of the reasons that LDME is much faster—it is free from the complexity involved in identifying and arranging the nodes for personalized compression based upon the influence of the target node.

**Figure 13:**
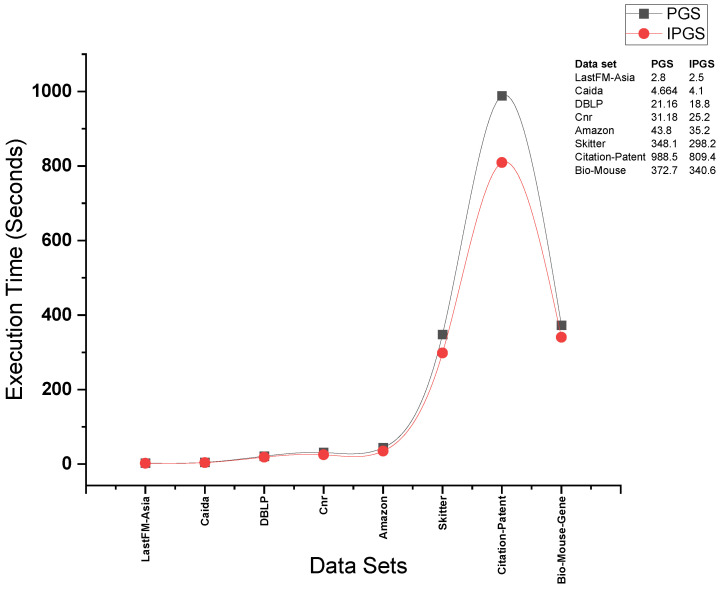
Results for execution time on complete size of each dataset.

Figure 13 presents the results for the execution times obtained by running PGS and IPGS on complete batch sizes. We observe that the running times of both of the algorithms show a better performance from IPGS in all the cases. The difference becomes clearer when the algorithms are applied to the Citation network dataset. This dataset has a significantly larger size; hence, it serves the purpose of our proposal.

**Figure 14:**
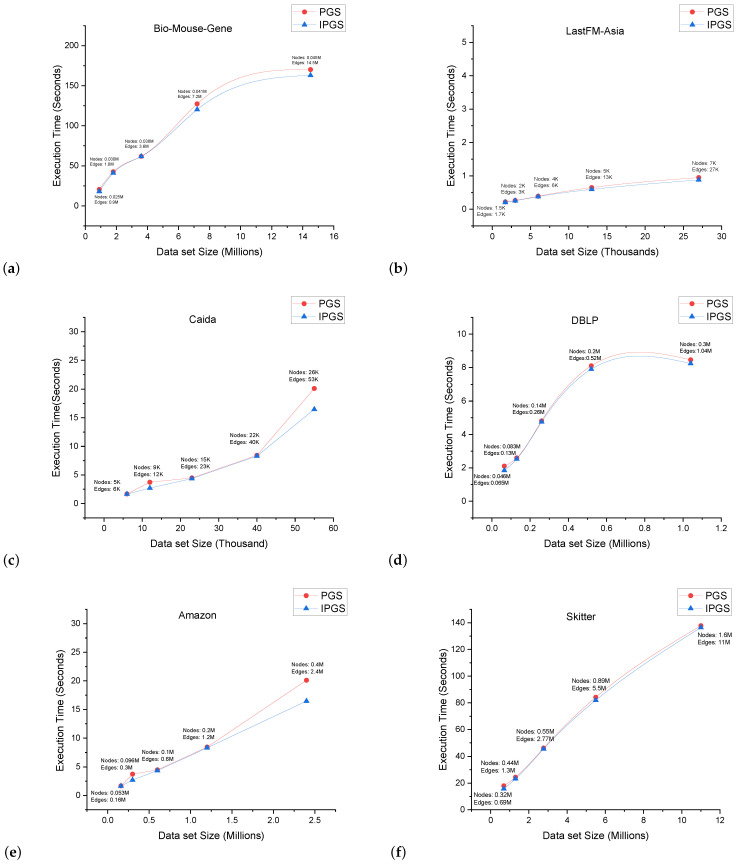

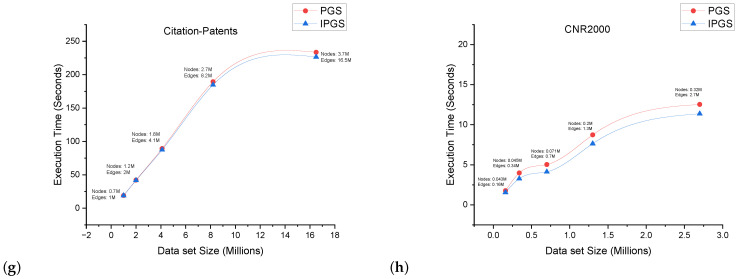
Execution time (in s) comparison of PGS and IPGS for different data sizes. The sub-figures (**a**–**h**) demonstrate the execution time on different datasets.The name of the dataset is shown at the top of each sub-figure.

Figure 14 demonstrates the results of the execution time comparisons for PGS and IPGS. In all of the experiments, we find that IPGS achieves a better performance than PGS. The Bio–Mouse–Gene, Skitter, Amazon, and Citation network datasets are much larger in size; yet, we find that the execution time achieved by both of the algorithms is reasonable. IPGS consistently outperforms PGS. The Citation network dataset is the largest dataset used in these comparisons, with 4 million nodes and 17 million edges. PGS took 17 min to run, while IPGS achieved the same task in 13 min. This trend persisted across all the other datasets as well.

For the execution time comparison, we analyzed PGS [43] and IPGS; in contrast, for the assessment of the compression ratio, we compare LDME [45], PGS [43], and IPGS.

### 5.4. Comparison of Compression Ratio

**Figure 15:**
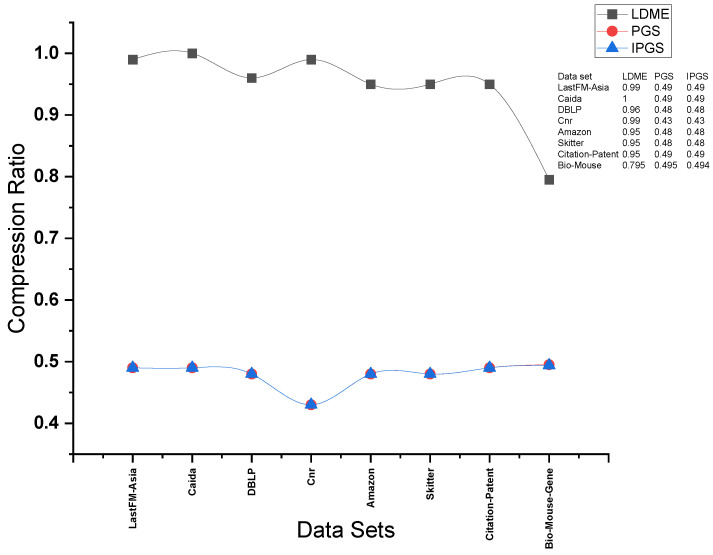
Results for compression ratio obtained on complete size of each dataset.

**Figure 16:**
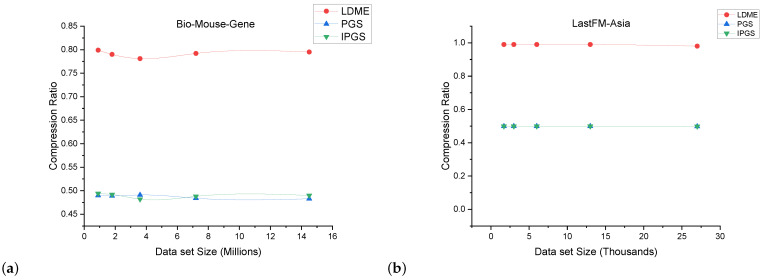

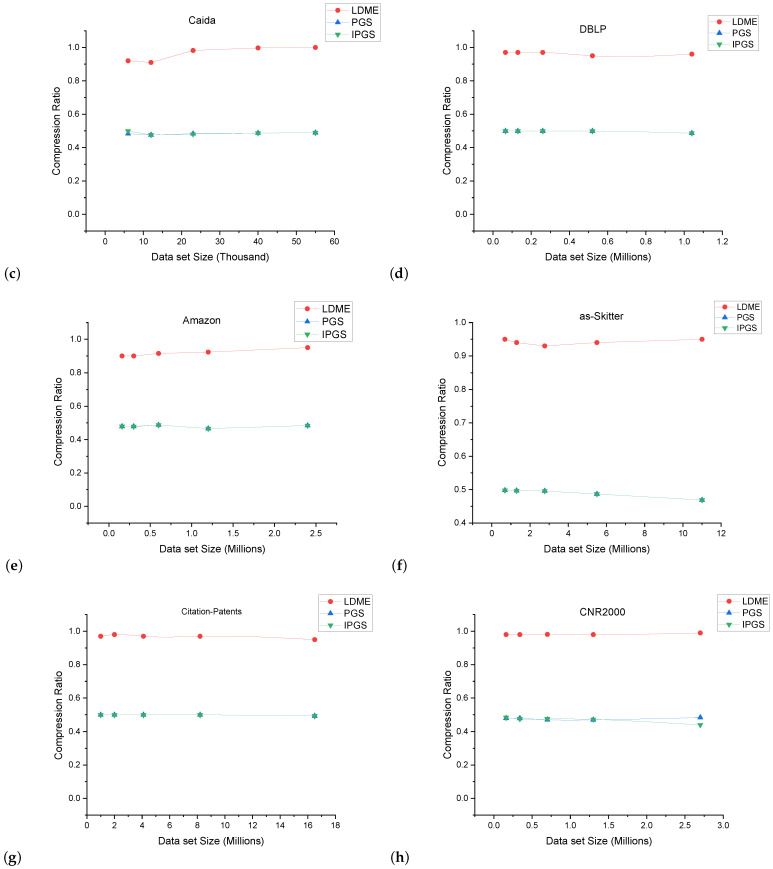
Comparison between LDME, PGS, and IPGS for compression achieved for different dataset sizes. The sub-figures (**a**–**h**) demonstrate the compression achieved on different datasets. The name of the dataset is shown at the top of each sub-figure.

The experimental results for the comparison of the compression ratio are presented in Figure 15 and Figure 16. The compression ratio is obtained using the formula in Equation (4). The size of the original input graph is computed using Equation (5). This formula calculates the size of the original graph based on the total number of edges (O_Edges) and the total number of nodes (O_Nodes) in the original graph. The formula involves multiplying the number of edges by a factor of 2 and then taking the logarithm base 2 of the total number of nodes. The formula in Equation (6) calculates the size of the summarized graph after summarization. This is based on the number of super edges (S_Edges) and the number of super nodes (_Nodes) in the summary graph, as well as the total number of original nodes (_Nodes) in the original graph. The formula involves multiplying the number of super edges by a factor of 2 and then taking the logarithm base 2 of the total number of super nodes. Additionally, it considers the contribution of the original nodes by multiplying their count with the logarithm base 2 of the total number of super nodes.

(4) $$(\mathrm{Compression}\;\mathrm{Ratio}=\frac{\mathrm{Size}\;\mathrm{of}\;\mathrm{Summary}\;\mathrm{Graph}}{\mathrm{Size}\;\mathrm{of}\;\mathrm{Original}\;\mathrm{Graph}})\;$$

(5) $$\mathrm{Size}\;\mathrm{of}\;\mathrm{Original}\;\mathrm{Graph}=\mathrm{O\_Edges}\times 2\times \log_{2}\left(\mathrm{O\_nodes}\right)\;$$

(6) $$\mathrm{Size}\;\mathrm{of}\;\mathrm{Summary}\;\mathrm{Graph}=\mathrm{S\_Edges}\times 2\times \log_{2}\left(\mathrm{S\_nodes}\right)+\mathrm{O\_nodes}\times \log_{2}\left(\mathrm{S\_nodes}\right)$$

The results of our evaluation for the comparison of the compression ratio are highly promising. For the largest network used for comparisons, i.e., the Citation network dataset, we achieved a compression ratio of 0.4; this is same as that achieved by PGS. Both PGS and IPGS achieved a compression of 60 percent. On the other hand, LDME achieved a compression ratio of 0.7, i.e., 30 percent less compression than PGS and IPGS. This substantial improvement in the compression ratio demonstrates the efficacy of our proposed solution. We have successfully optimized the compression ratio, resulting in a more compact representation of the input graph data while preserving the aspect of personalization. This advancement has significant implications for various applications that rely on the elegant storage and processing of graph data. The compression ratios of IPGS and PGS are almost the same because both methods follow a similar approach for node identification in the merging process

## 6. Conclusions

Research into biosensors to find solutions which will aid in the improvement of healthcare systems is highly important. We reviewed a number of studies exploring the datasets of biosensors and bioengineering from variety of angles. One research direction in this field is the investigation of the wealth of data through graph summarization. This is a process which aims to compress the size of the large graph that is input for efficient data mining and visualization. There are a number of general-purpose graph summarization techniques which produce a summary graph for an entire input graph, without focusing on the impact/existence of certain influential nodes in a given dataset. However, in this research, we present a personalized graph summarization approach which can extract pertinent information from graph data; thus, it can be tailored to individual preferences. This method allows users to extract and customize their analyses, leading to more focused and insightful outcomes. Our research introduces IPGS, a new algorithm which improves the execution time of an existing state-of-the-art approach (PGS), while achieving a similar compression ratio. IPGS is particularly useful in the domain of studying bioengineering because we can analyze the network structure of a particular entity (like a gene, phenotype, etc.) in an efficient and elegant manner. To ensure that our study is applicable in various domains, we considered scalability and efficiency as key considerations during the algorithm’s development; this allowed it to effectively handle various types of graph data using a single machine. Influenced by the high compression ratio achieved by PGS—which comes at the cost of a slower execution time—our algorithm, IPGS, provides a robust and efficient solution for personalized graph summarization, catering to the needs of diverse applications and datasets. Further research in this field may focus on exploring additional optimizations and extensions to enhance the algorithm’s capabilities and broaden its applicability across different domains.

## Data Availability

The datasets used in this work are publicly available and their details are provided in Section 5.1.
